# Effect of Geometrical Parameters of Microscale Particles on Particle-Stimulated Nucleation and Recrystallization Texture of Al-Si-Mg-Cu-Based Alloy Sheets

**DOI:** 10.3390/ma15227924

**Published:** 2022-11-09

**Authors:** Jonggyu Jeon, Sangjun Lee, Joonseok Kyeong, Seeun Shin, Heon Kang

**Affiliations:** 1 Department of Materials Science and Engineering, Yonsei University, 50 Yonsei-ro, Seodaemungu, Seoul 03722, Korea; 2 Materials Research Sector, Hyundai Mobis Co., Ltd., Yonhin-si 16891, Korea; 3 Department of Materials Science and Metallurgical Engineering, Sunchon National University, 255 Jungang-ro, Suncheon-si 57922, Korea; 4 Korea Institute of Industrial Technology, Siheung-si 15014, Korea

**Keywords:** Al-Si-Mg-Cu alloys, microscale particle, recrystallization behavior, texture, mechanical properties

## Abstract

The effects of the shapes (needle and round) and volume fractions (low and high) of microscale particles in Al-Si-Mg-Cu-based alloys on recrystallization behavior, texture evolution, mechanical properties, and formability are investigated. The recrystallized grain size decreases as the size and volume fraction of the particles decrease and increase, respectively, regardless of the particle shape. The investigated alloys with a relatively low volume fraction of 0.7 to 2.4 vol.% exhibit higher efficiency particle-stimulated nucleation (PSN) than alloys with a high volume fraction of 6.0 to 21.0 vol.%. This is because the interaction between the particles and dislocations cannot be greatly promoted when the volume fraction of the particles is large enough to form agglomerates. The sheets with round-shaped particles exhibit higher yield strength (YS) and elongation (EL) than sheets with needle-shaped particles. The improvement in YS is due to the combined effects of grain refinement and particle strengthening, and the EL is improved by reducing the probability of cracking at the tips of round-shaped particles. The sheets with round-shaped particles exhibit relatively higher average plastic strain ratio ($\bar{r}$) and planar anisotropy (∆r) than the sheets with needle-shaped particles, owing to the development of Goss {110}<001> or rotated-Goss {110}<110> orientations.

## 1. Introduction

There has been a considerable amount of research interest in Al alloys to help reduce the weight of automobiles and solve global environmental problems. In particular, heat-treatable Al-Si-Mg-Cu alloy sheets have received great attention for manufacturing the outer bodies of automobiles, such as hoods, side doors, and roof panels, owing to their low specific density, reasonable corrosion resistance, comparatively good formability, and high strength after the paint bake cycle [1,2]. However, the formability of Al-Si-Mg-Cu alloy sheets is lower than that of conventional steel sheets, making it difficult and expensive to manufacture automotive parts with complex shapes [3].

The recrystallization behavior and texture evolution, which are mainly determined by particle-stimulated nucleation (PSN), significantly affect the mechanical properties and formability of the final gauge sheet [4]. The highly strained regions in the vicinity of microscale particles serve as preferred nucleation sites for recrystallization, forming fine recrystallized grains with random orientations [5]. Numerous studies have shown that various microscale particles, such as eutectic Si, Mg_2_Si, Al_7_Cu_2_Fe, Al_2_CuMg, and β-Al(FeMnCr)Si particles, promote PSN to refine the recrystallized grains [6,7,8]. The microscale particles with a size suitable for PSN (>1 μm) easily induce non-uniform stored energy that drives the nucleation of recrystallized grains. When the average sizes of the microscale particles are similar, the increase in the volume fraction of microscale particles promotes the occurrence of PSN. Zhang et al. [8] reported that the Mn content increased the volume fraction of microscale β-Al(MnCrFe)Si particles, which could promote the formation of recrystallized grains. Particles with a relatively large aspect ratio have been reported to induce a large strain gradient compared to particles with a small aspect ratio [9,10], but their effect on recrystallization behavior has not been discussed in detail. Since geometrical parameters of the particles, such as the shape, size, and volume fraction, have complex effects on recrystallization behavior, texture evolution, mechanical properties, and formability, this requires further studies to clearly understand the complex effects of and expand the applicability of Al-Si-Mg-Cu alloy sheets.

In this study, four Al-Si-Mg-Cu-based alloy sheets with different shapes (needle and round) and volume fractions (low and high) of microscale particles were prepared by controlling the fabrication methods and process conditions. The effects of the shape, size, and volume fraction of the particles on recrystallization behavior, texture evolution, mechanical properties, and formability were systematically investigated.

## 2. Materials and Methods

**Figure 1:**
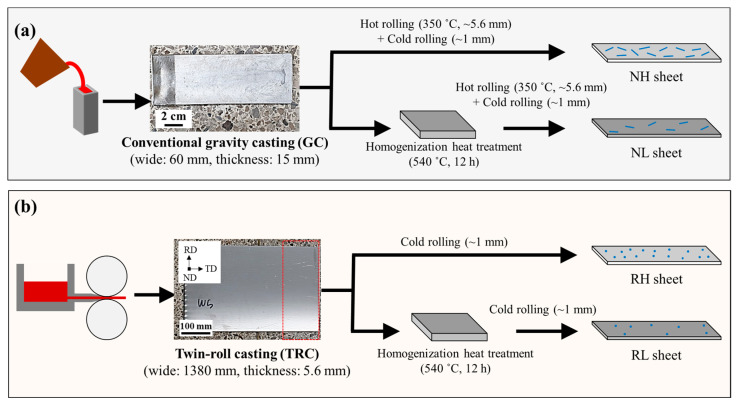
Schematic of the manufacturing method of NH, NL, RH, and RL sheets. The prepared GC and TRC alloys were each made into sheets using two different processes: (**a**) rolling without homogenization heat treatment and (**b**) rolling after homogenization heat treatment at 540 °C for 12 h.

Two methods of twin-roll casting (TRC) and gravity casting (GC) were used to prepare alloys to obtain round- and needle-shaped microscale particles, respectively, as shown in Figure 1. The TRC strip, whose width and thickness were 1380 mm and 5.6 mm, respectively, was manufactured by Choil Aluminum Co. (Gyeongsan-si, Korea) with a casting speed of 850 mm/min at 710 °C. The GC alloy was fabricated by adding alloying elements to the melt at 740 °C, followed by casting into a steel mold with a thickness of 10 mm. The GC alloy was hot-rolled up to 5.6 mm at 350 °C to achieve the same total reduction ratio in cold rolling as the TRC alloy. The chemical composition of the fabricated alloys was determined using optical emission spectrometry (SPECTROCHECK, Ametek Co., Berwyn, PA, USA). Table 1 presents the chemical compositions of the TRC and GC alloys. The alloys with a relatively low and high volume fractions of microscale particles were obtained by providing process conditions with and without homogenization heat treatment (at 540 °C for 12 h), respectively. Accordingly, the investigated alloys were named NH, NL, RH, and RL according to the shapes (needle and round) and volume fractions (high and low) of the microscale particles. The alloys with a thickness of 5.6 mm were cold-rolled for up to 1 mm with a reduction of 20%. The cold-rolled sheet was recrystallized at 540 °C for 30 min and then quenched with water.

The distribution and composition of the microscale particles were analyzed using a combination of scanning electron microscopy (SEM, JEOL JSM-7001F, Tokyo, Japan) and energy dispersive X-ray spectrometry (EDS, AMETEK Octane plus, Mahwah, NJ, USA). All specimens for SEM analysis were polished with up to 4000-grit SiC paper and then carefully polished using buffing and abrasion (Alumina suspension, 0.05 μm, Allied High Tech Products, Inc., Rancho Dominguez, CA, USA) with water. The crystallographic orientations of the specimens were evaluated using electron backscattered diffraction (EBSD, EDAX-TSL, Draper, UT, USA). The inverse pole figure (IPF) maps were observed with a step size of 3.5 μm. The orientation distribution functions (ODFs) were analyzed using orientation imaging microscopy (OIM) analysis software (TSL OIM analysis 7.3, EDAX Inc., Mahwah, NJ, USA). The software ImageJ (version 1.41o, National Institutes of Health, Bethesda, MD, USA) was used to calculate the size and volume fraction of the microscale particles. The average size and volume fraction of the microscale particles were calculated in three planes: rolling direction (RD), transverse direction (TD), and normal direction (ND).

Tensile tests were performed using an Instron-type tensile machine (Unitech^TM^, R&B, Daejeon, Korea) with a strain rate of 10^−3^ s^−1^ at 25 °C. Tensile specimens according to ASTM E8 were machined from the fabricated Al sheets. The yield strength (YS, 0.2% offset yield stress), ultimate tensile strength (UTS), elongation (EL), average plastic strain ratio ($\bar{r}$), and planar anisotropy (∆r) of the recrystallized sheets in the three directions of 0°, 45°, and 90° with respect to the RD were evaluated as the average of the three measurements.

## 3. Results and Discussion

**Figure 2:**
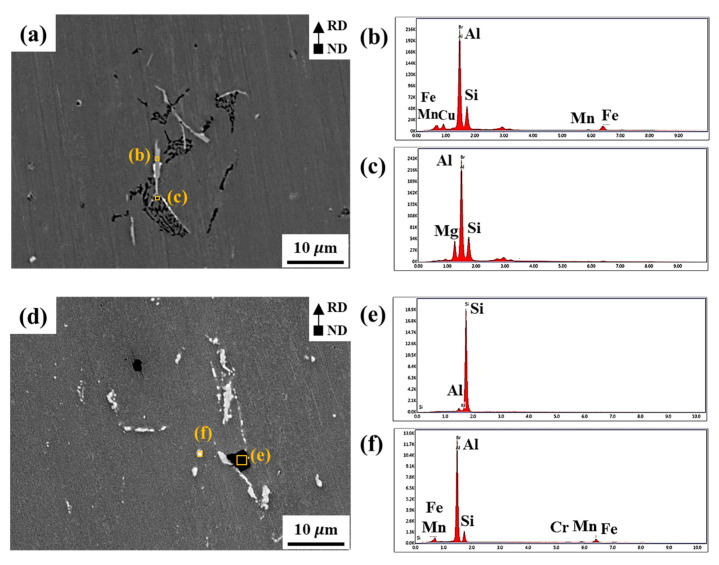
SEM images of (**a**) RH and (**d**) RL sheets, and (**b**,**c**,**e**,**f**) EDS spectrum of marked area marked in (**a**,**d**).

Figure 2a–f shows the SEM images and corresponding EDS results in the RH and RL sheets, respectively. Figure 2a–c show the needle-shaped β-AlFeSi and skeleton-shaped eutectic Mg_2_Si particles, which are consistent with the microstructures reported in other studies [11,12]. The Fe element has high solubility in liquid Al but extremely low solubility in solid Al, unintentionally forming Fe-containing intermetallic compounds [13]. The β-AlFeSi particles are mainly formed in Al-Si-Mg-based alloys due to Fe impurities in the raw Al with a purity of 99.8% [13,14]. After homogenization heat treatment, the majority of the eutectic Mg_2_Si particles were dissolved in Al matrix, but there are polygonal-shaped Si particles (Figure 2e) and round-shaped α-Al(FeCrMn)Si particles (Figure 2f). According to the reported results [15,16,17], a homogenization heat treatment promotes the transformation of needle-shaped β-AlFeSi particles into smaller round-shaped α-Al(FeCrMn)Si particles. Kuijpers et al. [18] reported that the β-AlFeSi particles were transformed into particles denoted as α-Al(FeMn)Si, α-Al_8_(FeMn)_2_Si, and α-Al_12_(FeMn)_3_Si. After homogenization heat treatment, the volume fraction of α-Al(FeCrMn)Si particles is relatively larger than that of β-AlFeSi particles [17,19]. Kuijpers et al. [18,20] suggested that α-Al(FeMn)Si nucleated on the basal face of β-AlFeSi particles; β-AlFeSi particles partially dissolve and then α-Al(FeMn)Si particles grow by consuming released Mn content. In addition, an increase of homogenization temperature can induce greater transformation of β-Al_5_FeSi particles into α-Al_8_(FeMn)_2_Si particles [16]. A high temperature can accelerate the dissolution of β-AlFeSi particles, promoting the formation of α-Al(FeCrMn)Si particles [21].

**Figure 3:**
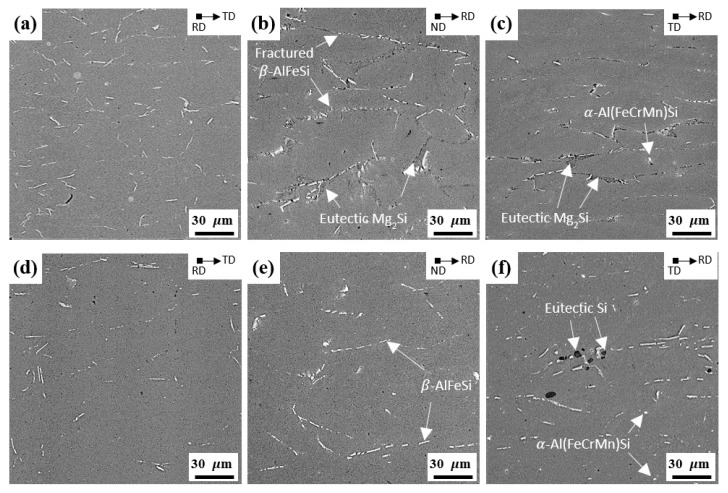
SEM images of (**a**–**c**) NH and (**d**–**f**) NL alloys with a thickness of 5.6 mm before cold rolling: (**a**,**d**) RD, (**b**,**e**) ND, and (**c**,**f**) TD.

**Figure 4:**
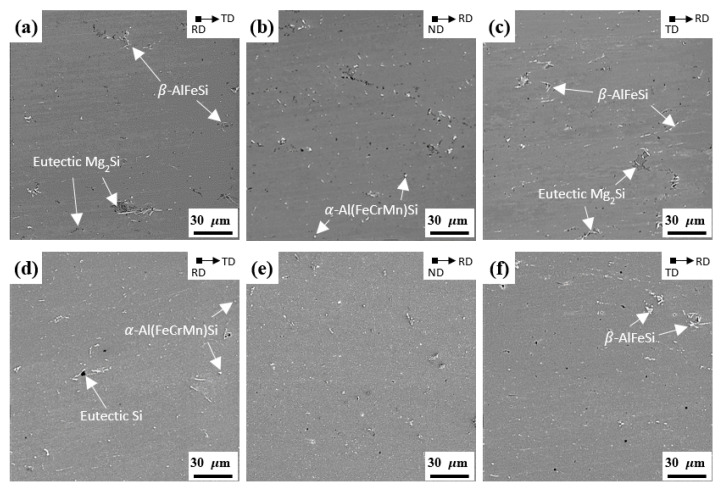
SEM images of (**a**–**c**) RH and (**d**–**f**) RL alloys with a thickness of 5.6 mm before cold rolling: (**a**,**d**) RD, (**b**,**e**) ND, and (**c**,**f**) TD.

Figure 3a–f and Figure 4a–c show the SEM images of the NH, NL, RH, and RL alloys before cold rolling, respectively. The NH and NL alloys show that the needle-shaped β-AlFeSi particles are crushed and aligned parallel to the RD, owing to the drag effect of the particles during hot rolling. On the other hand, the round-shaped particles of the RH and RL alloys are uniformly distributed with a relatively small size. The volume fraction of β-AlFeSi particles increased as the cooling rate during solidification decreased, whereas a high cooling rate can promote the formation of α-Al(FeCrMn)Si and suppress the formation of β-AlFeSi particles [15,22,23]. Therefore, the NH and NL alloys exhibit a large volume fraction of needle-shaped β-AlFeSi particles due to the slow cooling rate of the GC process. However, a large volume fraction of round-shaped particles was formed in RH and RL alloys, owing to the rapid solidification of the TRC process. The NH and RH alloys show the β-AlFeSi, α-Al(FeCrMn)Si, and eutectic Mg_2_Si particles, which are consistent with the other results [24]. After homogenization heat treatment (NL and RL alloys), the majority of the eutectic Mg_2_Si particles are dissolved in the Al matrix because of the high diffusivity of Mg in Al [25].

**Figure 5:**
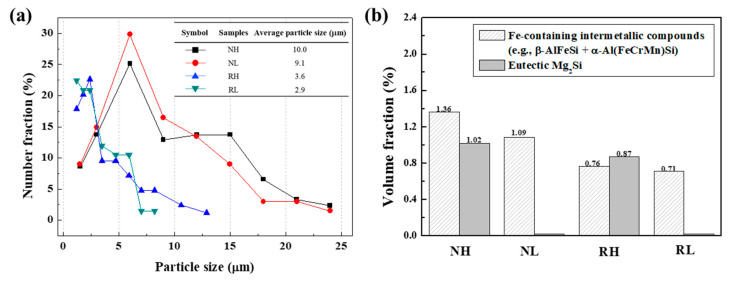
(**a**) Size distribution of the microscale particles in the NH, NL, RH, and RL alloys before cold rolling. The average sizes of the particles are interpolated in the figure. (**b**) Volume fractions of the Fe-containing intermetallic compounds and eutectic Mg_2_Si particles in the NH, NL, RH, and RL alloys before cold rolling.

Figure 5a shows the size distribution of the microscale particles before cold rolling. The average particle sizes measured for the NH, NL, RH, and RL alloys are 10.0, 9.1, 3.6, and 2.9 μm, respectively. The volume fractions of the Fe-containing intermetallic compounds and eutectic Mg_2_Si particles are shown in Figure 5b. The volume fractions of Fe-containing intermetallic compounds in NH, NL, RH, and RL alloys are 1.36, 1.09, 0.76, and 0.71 vol.%, respectively. The volume fractions of eutectic Mg_2_Si particles in the NH and RH alloys are 1.02 and 0.87 vol.%, respectively. The RH and RL alloys possess a relatively small size and volume fraction compared to the NH and NL alloys, owing to the rapid solidification of the TRC process. After homogenization heat treatment (NL and RL alloys), the needle-shaped β-AlFeSi particles are partially transformed into the round α-Al(FeCrMn)Si particles, reducing their average particle sizes [18,20]. In addition, the total volume fraction of the microscale particles in the NH and RH alloys are twice as large as those of the NL and RL alloys, respectively, owing to the presence of eutectic Mg_2_Si particles.

**Figure 6:**
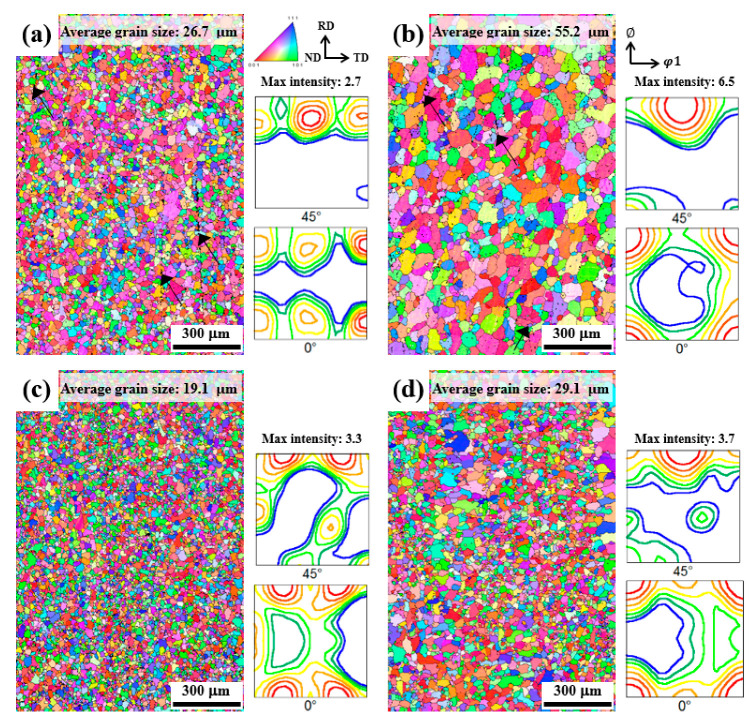
IPF maps and ODFs in the (**a**) NH, (**b**) NL, (**c**) RH, and (**d**) RL sheets recrystallized at 540 °C for 30 min. The microscale particles of the NH and NL sheets are crushed and aligned parallel to the RD, as indicated by the black arrows.

Figure 6a–d show the IPF maps and ODFs of the NH, NL, RH, and RL sheets recrystallized at 540 °C for 30 min, respectively. In the IPF maps, the grain boundaries are determined using a 15° misorientation criterion and are represented by solid lines. The black dots (indicated by black arrows) with a confidence index less than 0.1 correspond to regions where the system cannot specify any orientation, indicating the microscale particles. The NH and NL sheets exhibit a larger number of particles than the RH and RL sheets because of the relatively large size and volume fraction of the particles. The average grain sizes of the NH, NL, RH, and RL recrystallized sheets are 26.7, 55.2, 19.1, and 29.1 μm, respectively. The mechanism by which the recrystallized grain size varies significantly with the shape, size, and volume fraction of the microscale particles will be described later. In the ODFs, the strong cube {001}<100> orientation, commonly developed in the NL and RL sheets, results from the weak PSN, owing to the relatively low volume fraction of the particles. The RH and RL sheets develop Goss {110}<001> and rotated-Goss {110}<110> orientations, respectively.

**Figure 7:**
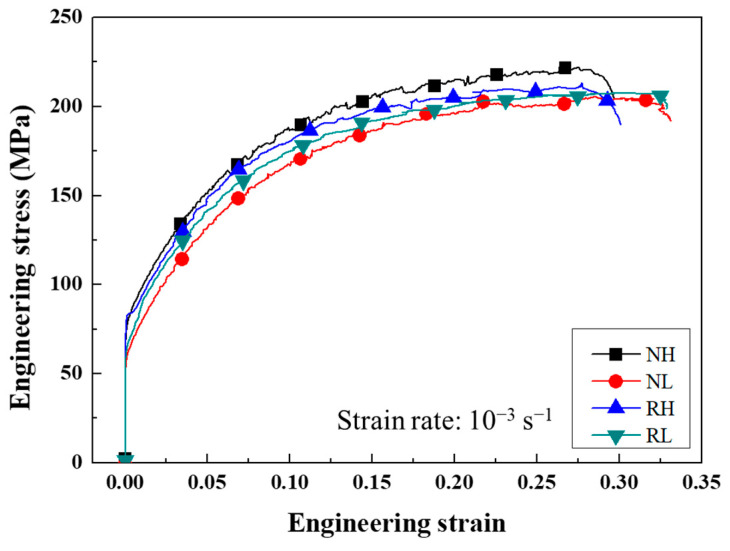
Engineering stress–strain curves in the RD of recrystallized sheets.

Figure 7 shows the engineering stress–strain curves of recrystallized sheets in the RD. Although the NH sheet exhibits a higher UTS than the other sheets, the highest YS is shown by the RH sheet. The RH sheet displays the discontinuous yielding phenomenon (Piobert–Lüders effect) due to fine recrystallized grains. This phenomenon, in which solute atoms act as obstacles to the dislocation movement, is more frequently encountered in fine-grained alloys [14].

The mechanical properties and formability of the recrystallized sheets corresponding to tensile directions of 0°, 45°, and 90° are summarized in Table 2. The RH and RL sheets exhibit a relatively higher YS than the NH and NL sheets, respectively, owing to the combined effects of grain boundary and particle strengthening. The grain boundary strengthening cannot significantly improve the strength in Al-Si-Mg-Cu alloys, owing to the relatively low Hall–Petch constant ($\sigma_{0}\;$ = 5.5 MPa and k  = 40 MPa μm^1/2^ [26]). In addition, the average EL of the NH and NL sheets is relatively lower than that of the RH and RL sheets because the needle-shaped particles easily cause the formation of cracks, owing to the stress concentration at the tips. The texture evolution and hence the r-value is highly dependent on the shape, size, and volume fraction of the microscale particles because the texture develops from the competition of nucleation at the cube bands, high-angle grain boundaries, and near-microscale particles [27,28]. The RH and RL sheets exhibit relatively higher $\bar{r}$ and Δr values than the NH and NL sheets, respectively, owing to the development of Goss {110}<001> or rotated-Goss {110}<110> orientations [29,30]. The Goss {110}<001> orientation is nucleated from high-angle boundaries formed by the strong interaction between dislocations and round-shaped particles with narrow inter-particle spacing [31,32]. In addition, the NH and RH sheets exhibit higher $\bar{r}$ and lower Δr values than the NL and RL sheets, respectively. This is because the more frequent occurrence of PSN in NH and RH sheets produces large numbers of grains with various orientations [33,34,35]. Thus, the maximum intensities of the NH and RH sheets in the ODFs are relatively smaller than those of the NL and RL sheets (Figure 6), which indicates the development of a relatively random texture. Furthermore, the NH and RH sheets show a rotated-cube {001}<110> orientation nucleated near microscale particles, whereas the NL and RL sheets show a strong cube {001}<100> orientation nucleated at the cube bands, owing to the weak occurrence of PSN [4,28,36]. The rotated-cube {001}<110> orientation exhibits a relatively higher $\bar{r}$ and lower Δr compared to the cube {001}<100> orientation [37].

**Figure 8:**
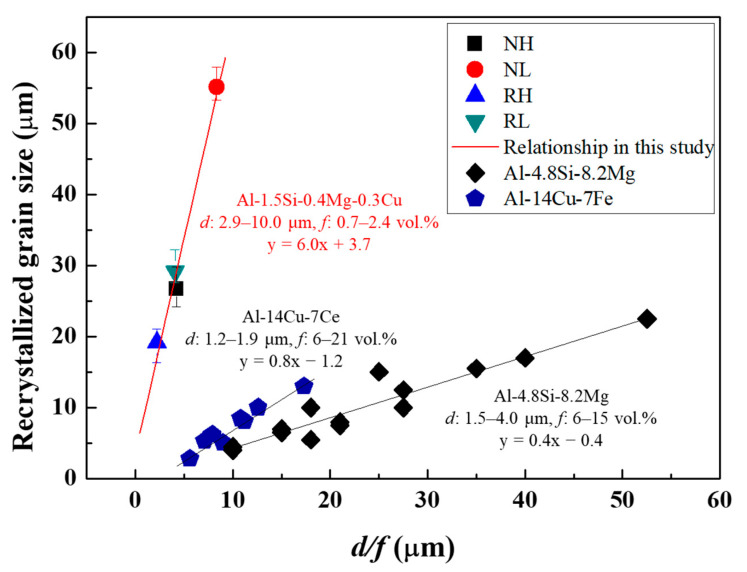
Dependence of the recrystallized grain size on d/f in the different Al alloys. Results for Al-4.8Si-8.2Mg and Al-14Cu-7Fe alloys were reported by Mikhaylovskaya, et al. [38].

The relationship between the recrystallized grain size and ratio of the size (d) to volume fraction (f) of the microscale particles is shown in Figure 8. Mikhaylovskaya, et al. [38] reported that the recrystallized grain size (D) is proportional to the d/f value, and it can be expressed as:

(1) $$D=k\;\left(\frac{d}{f}\right)+b,$$

where b is the theoretical minimum of the recrystallized grain size. k (slope of the linear relationship) is a constant related to the sensitivity on which the recrystallized grain size depends when the size and volume fraction of the microscale particles are changed. A relatively high k value indicates that small changes in the d/f values significantly alter the recrystallized grain size. The d/f values for the NH, NL, RH, and RL alloys are 4.21, 8.35, 2.21, and 4.08, respectively, which are linearly related to their recrystallized grain sizes regardless of the particle shape. Consequently, the k value of 6.0 obtained in this study is larger than the k values of 0.4 to 0.8 reported in Mikhaylovskaya’s study [38], which can be explained by the volume fraction of microscale particles. A large volume fraction of particles with a range of 6–21 vol.% can reduce the efficiency for PSN because the particles are more likely to agglomerate than maintain a proper distance. However, in this study, a relatively small volume fraction of the particles ranging from 0.7 to 2.4 vol.% can improve the efficiency for the occurrence of PSN (i.e., high k value). More specifically, it can be demonstrated by considering the concept of inter-particle spacing. The inter-particle spacing (λ) is estimated using the following equation [39]:

(2) $$\lambda =\;\frac{2}{3}d\left(\frac{1}{f}-1\right).$$

As the particle size decreases and their volume fraction increases, the inter-particle spacing decreases. The narrow inter-particle spacing causes strong interactions between the particles and dislocations by the Orowan looping model, forming a large strain gradient. Regions with large strain gradients near the microscale particles serve as preferred nucleation sites for recrystallization, refining the recrystallized grains [40,41]. However, when the volume fraction of microscale particles is large enough to form agglomerates, the interaction between the particles and dislocations (i.e., the occurrence of PSN) cannot be greatly promoted. Therefore, in this study, the Al-Si-Mg-Cu alloys with a low volume fraction in the range of 0.7 to 2.4 vol.% have a large k value of 6.0, owing to the high-efficiency PSN by uniformly distributed particles. Consequently, the k value can significantly vary when the range of volume fractions of the particles is different, even in a similar alloy system, as shown in Figure 8.

The number of PSN nuclei increases with increasing local non-uniform deformation in the particle deformation zone (PDZ) [42,43]. The particle shape has substantially different effects on the degree of local non-uniform deformation in the PDZ. The rod-shaped particles generated a larger local non-uniform deformation in the PDZ than the round-shaped particles. According to a previous study [43], a higher dislocation density is accumulated at the tip of rod-shaped Al_2_Cu particles than at that of round-shaped Al_6_Mn particles, leading to strong lattice rotation. In addition, the particles with a large aspect ratio form a larger strain gradient than the particles with a small aspect ratio [44]. However, in this relationship, the shape of the microscale particles in the initial state has little effect on the recrystallization behavior because the needle-shaped β-AlFeSi particles with extremely brittle properties are crushed and distributed during cold rolling (Figure 6a,b). Before the stress concentration at the tip of the needle-shaped particles exceeded a certain level, the particles were crushed to release the stress concentration. Consequently, the ratio of the size to volume fraction of the microscale particles is proportional to the recrystallized grain sizes regardless of particle shape.

## 4. Conclusions

The ratio of the size to volume fraction of the microscale particles is proportional to the recrystallized grain sizes. However, the shape of the particles does not affect the relationship significantly because the needle-shaped β-AlFeSi particles are crushed and distributed during cold rolling. As the microscale particle size decreases and their volume fraction increases, the narrower inter-particle spacing causes strong interactions between particles and dislocations, reducing the recrystallized grain size. When the volume fraction of microscale particles is large enough to form agglomerates, the interaction between the particles and dislocations cannot be greatly promoted. Therefore, the Al-Si-Mg-Cu alloys with a relatively low volume fraction ranging from 0.7 to 2.4 vol.% exhibit a large k value of 6.0, owing to the high-efficiency PSN by uniformly distributed particles. The RH and RL sheets exhibit higher average YS and EL than the NH and NL sheets, respectively. In addition, the RH and RL sheets exhibit relatively higher $\bar{r}$ and Δr values than the NH and NL sheets. This is because the Goss {110}<001> or rotated-Goss {110}<110> orientations develop at high-angle grain boundaries formed by the narrowly spaced round-shaped particles. The NH and RH sheets with a large volume fraction of the particles exhibit fine recrystallized grains with random orientations, owing to the more frequent occurrence of PSN, resulting in an increase and decrease in $\bar{r}$ and Δr, respectively.

## Figures and Tables

**Table 1:** Chemical compositions (in wt.%) of the Al-Si-Mg-Cu alloy sheets.

Samples	Si	Mg	Cu	Fe	Mn	Cr	Al
NH	1.51	0.40	0.30	0.18	0.10	0.05	Bal.
NL							
RH	1.53	0.41	0.29	0.16	0.09	0.06	
RL							

**Table 2:** Recrystallized grain size, YS, UTS, EL, $\bar{r}$ , and ∆r of the NH, NL, RH, and RL recrystallized sheets corresponding to the tensile directions of 0, 45, and 90° with respect to the RD.

Samples	Tensile Direction (°)	Grain Size (μm)	YS (MPa)	UTS (MPa)	EL (%)	r	$\bar{r}$	Δr
NH	0	26.7	78.8	219.9	29.9	0.63	0.66	−0.006
	45		75.8	226.5	30.1	0.66		
	90		78.4	224.6	29.2	0.68		
NL	0	55.2	61.8	210.9	32.0	0.61	0.58	−0.07
	45		66.4	211.4	28.9	0.55		
	90		66.3	216.6	29.6	0.63		
RH	0	19.1	80.4	210.7	32.2	0.58	0.69	−0.22
	45		80.8	207.9	32.5	0.80		
	90		81.1	211.4	29.6	0.58		
RL	0	29.1	73.7	210.8	31.9	0.91	0.66	0.29
	45		74.2	211.3	31.9	0.51		
	90		70.5	205.1	29.8	0.70		

## Data Availability

Data available in a publicly accessible repository.
